# Adiponectin Paradox in Alzheimer's Disease; Relevance to Amyloidogenic Evolvability?

**DOI:** 10.3389/fendo.2020.00108

**Published:** 2020-03-04

**Authors:** Masaaki Waragai, Gilbert Ho, Yoshiki Takamatsu, Ryoko Wada, Shuei Sugama, Takato Takenouchi, Eliezer Masliah, Makoto Hashimoto

**Affiliations:** ^1^Laboratory for Parkinson's Disease, Tokyo Metropolitan Institute of Medical Science, Tokyo, Japan; ^2^Department of Neurodegenerative Diseases, PCND Neuroscience Research Institute, Poway, CA, United States; ^3^Department of Physiology, Nippon Medical School, Tokyo, Japan; ^4^Institute of Agrobiological Sciences, National Agriculture and Food Research Organization, Tsukuba, Japan; ^5^Division of Neurosciences, National Institute on Aging, National Institutes of Health, Bethesda, MD, United States

**Keywords:** adiponectin, adiponectin paradox, chronic heart failure (CHF), Alzheimer's disease (AD), evolvability, antagonistic pleiotropy

## Abstract

Adiponectin (APN) is a multi-functional adipokine which sensitizes the insulin signals, stimulates mitochondria biogenesis, and suppresses inflammation. By virtue of these beneficial properties, APN may protect against metabolic syndrome, including obesity and type II diabetes mellitus. Since these diseases are associated with hypoadiponectinemia, it is suggested that loss of function of APN might be involved. In contrast, despite beneficial properties for cardiovascular cells, APN is detrimental in circulatory diseases, including chronic heart failure (CHF) and chronic kidney disease (CKD). Notably, such an APN paradox might also be applicable to neurodegeneration. Although APN is neuroprotective in various experimental systems, APN was shown to be associated with the severity of amyloid accumulation and cognitive decline in a recent prospective cohort study in elderly. Furthermore, Alzheimer's disease (AD) was associated with hyperadiponectinemia in many studies. Moreover, APN was sequestered by phospho-tau into the neurofibrillary tangle in the postmortem AD brains. These results collectively indicate that APN might increase the risk of AD. In this context, the objective of the present study is to elucidate the mechanism of the APN paradox in AD. Hypothetically, APN might be involved in the stimulation of the amyloidogenic evolvability in reproductive stage, which may later manifest as AD by the antagonistic pleiotropy mechanism during aging. Given the accumulating evidence that AD and CHF are mechanistically overlapped, it is further proposed that the APN paradox of AD might be converged with those of other diseases, such as CHF and CKD.

## Introduction

**Figure 1:**
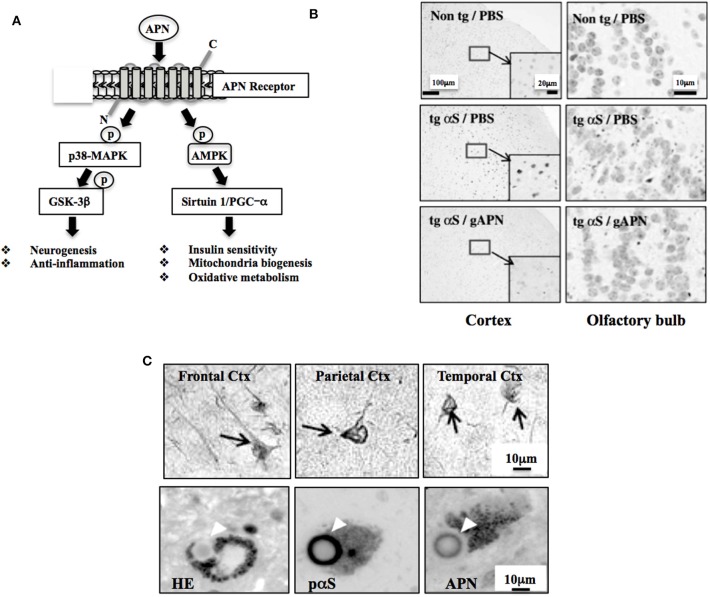
Neuroprotective and neurodegenerative effects of APN. **(A)** Schematic of APN signaling pathway. AMPK regulates various intracellular signaling molecules, such as sirtuin and PGC-1α, leading to stimulation of insulin sensitivity, mitochondrial biogenesis, and oxidative metabolism. APN also activates p38-MAPK, inhibiting GSK-3β activity, leading to stimulation of neurogenesis and suppression of neurodegeneration. Modified from Waragai et al. (1) with permission. **(B)** APN ameliorates neurodegeneration in a mouse model of α-synucleinopathies. Globular APN [gAPN, 0.1 mg/ml in 10 μl phosphate buffered saline (PBS)] or PBS alone (10 μl) was given intranasally to αS tg mice (male, 3 months old) or wild-type littermates every 3 days for 2 months. Brains were analyzed immunohistochemically (anti-phospho-αS). Representative images of the cortex and olfactory bulb are shown. Insets show a higher magnification of the cortex. Reprinted from Sekiyama et al. (20) with permission. **(C)** Involvement of APN in the pathogenesis of neurodegenerative diseases, including AD and α-synucleinopathies, including Parkinson's disease and dementia with Lewy bodies. Immunohistochemical staining using a polyclonal anti-APN C-terminal antibody showed strong immunoreactivity of APN in inclusion bodies; neurofibrillary tangles of frontal, parietal and temporal cortex in AD brain (upper 3 panels) and Lewy bodies of α-synucleinopathies (lower 3 panels). Modified from Waragai et al. (8) (upper 3 panels), and Sekiyama et al. (20) (lower 3 panels) with permission. The lower figures; HE (left) and histochemistry (middle and right), were prepared from the consecutive sections.

Adiponectin (APN) is a multifunctional adipocytokine that is involved in diverse biological functions, including sensitization of the insulin receptor signaling pathway, mitochondria biogenesis, oxidative metabolism, neurogenesis, and suppression of inflammation (Figure 1A) (1). APN is produced mainly in adipose tissues (2). Multiple oligomerization of the 28 kDa monomer of APN exhibits different biological properties (2). The effects of APN are mediated through APN receptors; Adipo-R1 and -R2, and their downstream the signaling molecules, such as AMP-activated protein kinase, p38-MAPK and GSK-3β, sirtuin 1 and PGC-α (3), leading to modification of transcription.

Accumulating evidence suggests that APN may be beneficial for the metabolic disorders, including obesity and type II diabetes mellitus (T2DM). Because these diseases are characterized by hypoadiponectinemia, it is predicted that decreased function of APN might be attributed to the metabolic disorders. In contrast, APN is detrimental in other chronic disorders, such as chronic heart failure (CHF) and chronic kidney disease (CKD), in which the increased APN in plasma has been characterized (4). Although APN is protective for cardiovascular cell function (5), hyperadiponectinemia is well-correlated with the severity of circulatory diseases, including CHF and CKD (4). Such a phenomenon is called APN paradox, the mechanism of which is poorly understood.

Similarly, APN has also been characterized by protective and toxic dual functions in the nervous system. Despite of cell- and animal-based studies showing that APN was protective, APN was correlated with the severity of amyloid accumulation and cognitive decline in the elder population (6). Furthermore, hyperadiponectinemia was observed in Alzheimer's disease (AD) similar to CHF and CKD (7). Moreover, APN was sequestered by phospho-tau into the neurofibrillary tangle in the postmortem AD brains (8). Thus, these results indicate that the risk of AD might be enhanced by APN. Therefore, better understanding of the APN paradox in AD might be important from both mechanistic and therapeutic viewpoints.

In this paper, we discuss our hypothetical view that APN might have a critical role in stimulation of amyloidogenic evolvability in the reproductive stage, which may later manifest as APN-stimulation of AD by the antagonistic pleiotropy mechanism during aging. Given the accumulating evidence that AD and CHF might be considerably overlapped in their pathologies, it is further predicted that APN paradoxes in both disease might be interactive.

## Neuroprotective and Anti-Neurodegenerative Activities of APN in Experimental Models

### Diverse APN Actions in the Nervous System

Expression of the receptors of APN; Adipo-R1 and -R2, are both abundant in the hypothalamus, particularly in the paraventricular hypothalamus and the arcuate nucleus (9). Consistent with these results, intracerebroventricular administration of APN decreased body weight, mostly through stimulating energy expenditure in a mouse model of T2DM (10). Thus, APN might regulate energy balance and metabolism (11).

Beyond energy regulation, APN might be involved in other functions in the nervous system. For example, APN was neuroprotective against cytotoxicities caused by amyloid β (Aβ) and MPP+ *in vitro* (12, 13). *In vivo*, APN protected against kainic acid-induced excitotoxicity in hippocampus in mice brains (14). Notably, APN might regulate neurogenesis. In support of this notion, APN was shown to stimulate proliferation of adult hippocampal neural stem/progenitor cells through signaling cascades such as p38 mitogen-activated protein kinase/glycogen synthase kinase 3β/β-catenin (15). Furthermore, it was shown that physical exercise-induced hippocampal neurogenesis was mediated by APN (16). Moreover, APN was neurotrophic for dendritic arborization and spinogenesis in the dentate gyrus in mice brains (17).

In addition to the effects of APN on neuroprotection/neurogenesis, APN may be critical in suppression of neuroinflammation. It has been shown that APN might normalize the imbalance between M1 and M2 microglia (18), whereas globular APN was shown to induce a pro-inflammatory response in astrocytes (19). Collectively, further investigations are warranted to evaluate the *in vivo* effects of APN on microglia and astrocytes.

### Neurodegeneration Is Ameliorated by APN in Mice

Given that the APN functions is diverse in the nervous system, it is curious to know if APN might be therapeutic for neurodegenerative disorders. Indeed, various neuropathological features, such as protein aggregation and impaired motor activity were ameliorated by treatment of APN in a mouse model of α-synucleinopathies (Figure 1B) (20). Subsequently, osmotin, a plant homolog of APN, was shown to attenuate Aβ42-induced neurotoxicity and tau hyperphosphorylation in the hippocampus in mice brains (21). Moreover, a recent study showed that aged APN-knockout mice had developed characteristics of an AD-like pathology associated with dysregulation of insulin receptor signaling (22).

## APN Stimulates Neurodegeneration in Human Brain

Recent cohort study of Mayo clinic showed that upregulation of plasma APN was significantly correlated with the severities of amyloid accumulation and cognitive decline in the elder population (6), indicating that APN might enhance the risk of AD. The results are surprising since the risk for AD and vascular dementia is increased by metabolic syndrome, such as T2DM, obesity, and atherosclerosis, and hypoadiponectinemia is a well-characterized feature of these metabolic diseases (4).

### Increased Level of Plasma APN in AD

To date, many clinical studies in AD have shown that there might be a positive correlation between APN and AD disease progression. A pilot study by Une and colleagues showed that the levels of plasma APN were significantly higher in both mild cognitive impairment (MCI) and AD compared to those in normal controls, and that the plasma levels of APN were correlated with cerebrospinal-fluid levels of APN (7). Similarly, the Framingham Heart prospective study (840 dementia-free elderly participants 299 men and 541 womes) showed that elevated APN was a predictor for AD and other types of dementia (23), whereby it was noted that the increase of plasma APN in AD was significant in women, but not in men (23). Increased levels of plasma APN in AD were confirmed by other studies (8, 24), whereas the lower plasma APN was also found (25). Although the reasons for the different results are elusive, it is possible that the APN level might be affected by various factors, including concurrent diseases or states that also affect APN levels. Furthermore, given that the function of the dissociated APN may be distinct from that of trimeric APN because of the different conformation (3), the discrepancy of different results from population studies might be affected by the handling of the samples or the antibody (3).

### Association of Altered Plasma APN With Neurodegenerative Pathology

Of a particular interest, the result of large cohort study of aging and dementia (*n* = 535, aged ≥ 70 years without dementia) conducted by the Mayo Clinic Study of Aging showed that higher plasma APN levels were correlated with imaging data for hippocampal and cortical volumes, positron emission tomography and cognitive deficits. Thus, the results suggest that higher APN predicts neurodegeneration and cognitive decline in aging (6). Notably, these results were significant in women but not in men, consistent with the results of the Framingham Heart Study (23). Considering that plasma APN in women is higher compared to that in men and the risk of AD is higher in women than in men, it is predicted that the gender difference of APN might in some parts contribute to the risk of AD.

### Accumulation of APN in Inclusion Bodies in Neurodegeneration

Consistent with the view that APN might be involved in the neurodegenerative pathogenesis, histopathological analyses of the autopsy brain of AD revealed that APN was sequestered by tau into the neurofibrillary tangles (Figure 1C upper) (8). Similarly, APN co-localized with Levy bodies in the brain of dementia with Lewy bodies (Figure 1C lower) (20). Together with the plasma data regarding APN, it is likely that increase of APN expression may be correlated with the development of neurodegenerative diseases, including AD and Parkinson's disease (PD).

## Mechanism of the APN Paradox in AD

Currently, the mechanism of hyperadiponectinemia in AD is unclear. In this regard, evolutionary biology might provide an effective viewpoint. We previously discussed that hyperadiponectinemia in AD might be a compensatory feedback to the decreased activity of insulin/IGF-1 receptor signaling pathway during the neurodegenerative conditions (1). As the disease progresses, APN might be increased and sequestered by tau, leading to neurotoxic protein aggregation in the brain of AD (1, 8). An alternative and non-exclusive possibility is that misfolding of APN might downregulate the insulin/APN signal transduction network, resulting in the decrease of neurotrophic and neuroprotective activities. Thus, it is predicted that alteration of APN may lead to synaptic loss and neuronal cell death in AD. Considering, however, that the sensitization insulin receptor signaling by APN may be evolutionally beneficial, such a compensatory mechanism may be only effective during the reproductive stage a viewpoint of evolutional biology.

### APN Stimulates Amyloidgenic Evolvabiliy?

**Figure 2:**
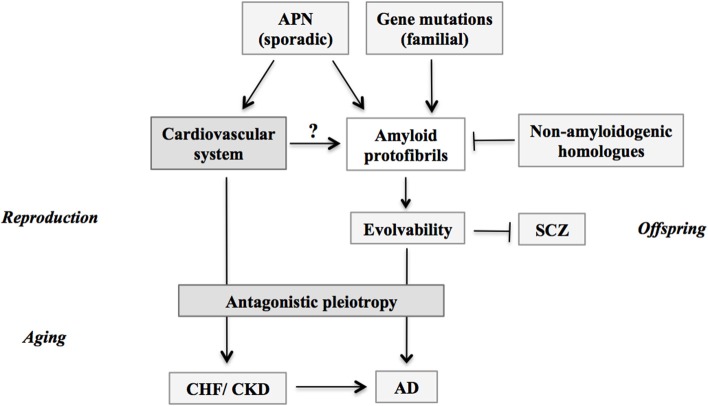
Schematic; stimulation of evolvability by APN and manifestation as diseases. APs protofibrils might be involved in the stress resistance, namely hormesis, in parental brain. Furthermore, by virtue of the information carried by the transmission of APs protofibrils in reproduction, offspring can cope with the forth-coming stresses in the brain to escape from neurodevelopmental diseases such as schizophrenia. Thus, the APs protofibrils may confer evolvability which is evolutionally beneficial. However, the evolvability of APs protofibrils may increase the risk of AD through the antagonistic pleiotropy. Amyloid protofibrils/evolvability may be stimulated by missense mutations of genes in familial AD, and by APN in sporadic AD, but suppressed by non-amyloidogenic homologous proteins such as β-synuclein. Given the effects of CHF and CKD to increase the risk of AD in aging, these phenomena might be attributed to the stimulatory effect of APN on amyloidogenic evolvability through cardiovascular system in the reproductive stages. APN, adiponectin; CHF, chronic heart failure; CKD, chronic kidney disease; AD, Alzheimer's disease; SCZ, schizophrenia.

As insulin resistance leads to hyperinsulinemia in metabolic disorders, it is probable that APN resistance could in some parts play a role for the hyperadiponectinemia in AD. If the APN resistance is an only pathological phenomenon, it should have been selected out during evolution. In fact, it has been shown that insulin resistance is not only pathological, but may provide evolutionary advantages through physiological functions. For instance, insulin resistance may play an important role in various pathophysiological states such as starvation, immune activation, growth and cancer (26). In the similar context, one may wonder if there might be some beneficial actions of APN during developmental/reproductive stages, which might manifest as AD and related diseases through the antagonistic pleiotropy mechanism in aging. In this regard, evolvability could be related. Based on the analogy with evolvability of yeast prion (27–29), we recently proposed that evolvability of amyloidogenic proteins (APs), including Aβ and α-synuclein (αS), might be physiologically important in human brain exposed to multiple stressors, such as hyperthermia, physical stress, kindling and oxidative stress (30). More precisely, the diverse β-sheet structures of the protofibrillar forms of APs might confer the stress-resistance, namely hormesis, against the diverse stresses in parental brains, which may be transmitted to offspring through germ cells (30, 31). Mechanistically, we speculate that αS, a monomer of which is unstable due to its intrinsically disordered nature (32), might become more stable through oligomerization, leading to formation of diverse strains of protofibrils. Such stable αS protofibrils may be feasible for transgenerational transmission to the offspring. By virtue of the stress information derived from parental brains, offspring's brain can cope with forth-coming stresses, otherwise leading to onset of neurodevelopmental diseases, such as schizophrenia (33). Thus, evolvability of APs might be interpreted as the inheritance of acquired characteristics against environmental stresses. On the other hand, neurodegenerative diseases including AD may manifest in parent's brain through the antagonistic pleiotropy mechanism in aging (31). Although the regulation of evolvabiliy is unclear, it is assumed that stimulation of evolvability would be beneficial. We therefore hypothesize that APN might be critical as a stimulator of amyloidogenic evolvability in developmental/reproductive stages, which may later manifest as AD through the antagonistic pleiotropy mechanism in aging (Figure 2). Thus, dual actions of APN may be attributed to the antagonistic pleiotropy of the APN-stimulation of evolvability.

### Modification of APN Stimulation on Amyloidgenic Evolvabiliy

Notably, globular APN is structurally similarity with tumor necrosis factor-α (TNF-α) (34). Accumulating evidence suggests that neuroinflammation might be a double-edged sword (35). On one hand, neuroinflammation might help to protect against various neuronal injuries, such as infection, physical insults and toxic chemicals during the reproduction period (35). On the other hand, dysregulated neuroinflammation may result in production of increased levels of pro-inflammatory cytotokines, such as TNF-α, leading to exacerbation of neurodegenerative diseases (36). Thus, it is possible that TNF-α and its receptors could cooperate with APN in both evolvability in reproduction and neurodegeneration in aging.

It is also possible that the effect of APN on evolvability might be modulated by various factors, including steroid hormones and cytokines. In support of this view, APN actions were stimulated through a cross-talk with estrogen receptor signaling pathway in breast cancer cells (37). Conversely, TNF-α was shown to impair APN signaling, mitochondrial biogenesis, and myogenesis in primary myotubes cultures (38).

### Experimental Approach

The effect of APN on evolvability could be evaluated using transgenic (tg) mice model of neurodegenerative diseases. For instance, it was recently shown that Aβ42 expression increases host survival in a Herpes simplex virus type-1 encephalitis in AD tg mouse model (39). It is possible that this infection model could be a good model to evaluate this issue. According to our theory, it is predicted that the offspring derived from the AD tg mice crossed with tg mice overexpressing APN in the brain, might be more resistant compared to those derived from the single tg of AD. In contrast, the offspring born from the AD tg mice crossed with APN-knock-out mice may be vulnerable. Distinct from evolvabilty that is a phenomenon in the reproduction stage, an antagonistic pleiotropy is in the post-reproductive senescence, which is specific to human (31). Therefore, mice may be not appropriate to investigate the post-reproductive senescence.

### Aβ and APN; Evolutionally Beneficial?

Finally, if Aβ and APN stimulate neurodegeneration, why these phenomena have not been selected out during evolution? It is unlikely that low evolutionary selection pressure in senescence may be attributed to the persistence of Aβ in the vertebrate genome (40). Thus, one may predict that there might be some beneficial functions for Aβ and other APs. Since the cooperation of APN with Aβ in evolvability is supposed to be beneficial, this could explain why the deteriorative actions of Aβ and APN paradox in aged brain have been persistent in evolution.

## APN Paradox in Other Chronic Diseases; Relevance to Evolvability?

APN paradox was primarily described for aging-associated chronic diseases, such as CHF and CKD. Notwithstanding its salutary effects on glucose metabolism, inflammation, and several atherosclerotic processes shown by experimental results, APN exhibits a deleterious role on both all-cause and cardiovascular mortality in CHF (41). Furthermore, patients with CKD are subjected to an increased cardiovascular risk associated with the APN paradox (42). So far, little has been known about the mechanism of APN paradox in these diseases.

Considering that the average onsets of CHF and CKD are in 60s (43, 44), the view of the natural selection would not support the idea that APN might be upregulated as a compensatory feedback to the reduced activity of insulin/IGF-1 receptor signaling pathways under the pathological conditions. As discussed earlier, it is naturally predicted that there might be some important physiological effects of APN during reproductive stage, which might manifest as diseases through antagonistic pleiotropy in the post-reproductive senescence. Although amyloid pathology is not associated, it is intriguing to speculate that a similar concept regarding the antagonistic pleiotropy of APN-action in the reproductive stage could be applicable to other chronic diseases with APN paradox, including CHF and CKD (Figure 2).

Then, what is the physiological effects of APN in reproduction, which later manifest as chronic diseases through the antagonistic pleiotropy in aging? In relation to this issue, it is worth noting that CHF and AD may be pathologically overlapped. Indeed, it has been proposed that CHF might be a risk factor for AD (45). Mechanistically, decreased cerebral blood flow due to CHF may result in the dysfunction of the neurovascular unit and an energy crisis in neurons. This may cause the impaired clearance of Aβ and hyperphosphorylation of tau, resulting in neurodegeneration featured with the formation of senile plaques and neurofibrillary tangles. Furthermore, antihypertensive drugs targeting renin-angiotensin system might attenuate incidence of AD and slow down cognitive decline in patients with AD (46).

The overlapping pathology was also described for CHF and PD (47). Thus, further prospective studies are warranted to confirm these intriguing findings. One possible mechanism accounted for the overlapping pathology of CHF and CKD with AD in aging might be the antagonistic pleiotropy of the stimulation of amyloidgenic evolvability by the circulating system in reproduction (Figure 2). Such a speculation may be reasonable providing that the circulation may be critical for transmission of amyloid protofibrils, especially transgenerational transmission from parent to offspring via germ cells (30). Taken together, it is predicted that the APN paradoxes of CHF and CKD might be attributed to the stimulation of amyloidogenic evolvability (Figure 2).

## Therapeutic Implication

Given the overlapping pathologies, including impairment of the insulin signaling, between T2DM and AD, several T2DM-approved drugs have been or are now tested in preclinical and clinical settings for AD (48–54). However, the APN paradox, such a unique phenomenon of APN action in AD, may require a novel therapeutic strategy that is distinct from previous therapy for metabolic synmdrome.

### Differential Therapy Strategy of APN

Given that the metabolic diseases are risk factors of AD, expression and activity of APN might be decreased in the pre-symptomatic stage of AD. Therefore, either APN receptor agonist or restoration of the APN expression may be effective for protection of AD. If APN stimulates amyloidogenic evolvability, then increased APN would be beneficial for offspring.

As for the APN receptor agonist, synthetic small-molecule AdipoRon was isolated by screening the compound library (55). Subsequently, AdipoRon was shown to improve metabolism in various tissues, including liver, skeletal muscle and adipose tissue, and to exert anti-diabetic effects at the organism level, while it normalizes a shortened lifespan associated with obesity. Thus, it is of interest to determine whether AdipoRon may be useful in the pre-symptomatic stage of AD.

On the other hand, up-regulation of APN may be detrimental in the symptomatic stage. Therefore, either APN antagonist or reduction of APN mRNA (e.g., antisense mRNA and miRNA) may be a differential therapy strategy. Since the trade-off effects may be concerned, it is predicted that the switching timing of the differential therapy strategy might be critical. If alteration of APN is obscure, combination of other biomarkers such as dipeptidyl peptidase 4 might be effective (56).

### Antagonistic Pleiotropy May Be a Therapy Target

Supposing that evolvability stimulated by APN might be manifest as AD through the antagonistic pleiotropy in aging, it is predicted that an attractive alternate therapy strategy might focus on the antagonistic pleiotropy mechanism (Figure 2). In this regard, it is of note that the result of genome wide association study revealed that 2q22.3, corresponding to the genes of TGFβ/activin receptor, linked with risks of coronary heart disease, CHF, stroke, T2DM, cancer, neurodegenerative diseases, and mortality, suggesting that these serine/threonine receptor signaling pathways might be relevant to the antagonistic pleiotropy (57). Therefore, it is predicted that modifying the TGFβ/activin receptor-signaling pathways could be therapeutically effective for aging-associated neurodegenerative diseases (58).

## Conclusion

In summary, it is intriguing to note that the APN paradox is commonly observed in aging-associated chronic diseases, including neurodegenerative diseases and circulating diseases. Thus, APN may be regarded as a major player in the new field of geroscience. At present, the mechanism of the APN paradox is elusive. Given that amyloidogenic evolvability in the reproduction may be manifested as AD through the antagonistic pleiotropy mechanism in aging, it is speculated that APN paradoxes in other chronic diseases, including CHF and CKD, might be attributed to the stimulation of the amyloidogenic evolvability (Figure 2).

Since APN actions are complicated, depending on the life-stages, it is not easy to conceive that APN might become a therapeutic target. Instead, APN could be useful as a biomarker of AD as well as other aging-associated chronic diseases. For this, it is possible that APN may be combined with other disease-specific molecules in aging. However, considering that the APN paradox occurs in the post-reproduction senescence that is a human-specific phenomenon, it is unlikely that rodents are appropriate as a model system to investigate this issue of the APN paradox. Thus, there being certain difficulties involved, further investigations are definitely warranted for better understanding of the role of APN in human aging.

## Data Availability Statement

All datasets generated for this study are included in the article.

## Author Contributions

MH and MW conceived the study. MH and GH wrote the paper. All authors have read and approved the manuscript.

### Conflict of Interest

The authors declare that the research was conducted in the absence of any commercial or financial relationships that could be construed as a potential conflict of interest.

## Glossary Style Box

Evolvability; Evolvability is the ability of a population of organisms to not merely generate genetic diversity, but to generate adaptive genetic diversity, and thereby evolve through natural selection (59). Namely, evolvability is defined as the capacity of a system for adaptive evolution.

Antagonistic pleiotropy; In 1957, George Williams proposed that animals possess genes that enhance fitness early in life but diminish it in later life and that such genes can be favored by natural selection because selection is stronger early in life even as they cause the aging phenotype to emerge (60). Thus, antagonistic pleiotropy of aging is the genetic trade-off between early life fitness and late life mortality.
